# Enhanced Apoptosis of Monocytes from Complication-Free Juvenile-Onset Diabetes Mellitus Type 1 May Be Ameliorated by TNF-***α*** Inhibitors

**DOI:** 10.1155/2014/946209

**Published:** 2014-06-02

**Authors:** Jolanta Myśliwska, Monika Ryba-Stanisławowska, Marcin Smardzewski, Bartosz Słomiński, Małgorzata Myśliwiec, Janusz Siebert

**Affiliations:** ^1^Department of Immunology, Medical University of Gdańsk, Ulica Dębinki 1, 80-211 Gdańsk, Poland; ^2^Academic Clinic of Pediatrics, Hematology, Oncology and Endocrinology, Medical University of Gdańsk, Ulica Dębinki 7, 80-211 Gdańsk, Poland; ^3^Department of Family Medicine, Medical University of Gdańsk, Ulica Dębinki 2, 80-211 Gdańsk, Poland

## Abstract

Diabetes mellitus type 1 is associated with an enhanced apoptosis of different cells and tissues, accelerating occurrence of diabetic microvascular complications. The aim of our study was to determine spontaneous apoptotic potential of the monocyte subsets in juvenile-onset complication-free diabetes mellitus type 1 and to compare them with the corresponding values of the healthy. Moreover, we wanted to assess effects of TNF-R1 blocking agents and those of general TNF-*α* blocker (Infliximab) on spontaneous apoptosis of monocytes. Sixty randomly selected DM1 patients (14.5 ± 3.2 years) and 30 healthy (13.5 ± 2.8 years) volunteers were enrolled in the study. Our results indicate that three monocyte subsets are distinguishable in the groups of young diabetic patients and the healthy, similarly to in the blood of adults. DM1 patients were characterized by higher values of apoptotic monocytes than the healthy. The manipulation with drugs inhibiting TNF-R1 expression diminished the pool of CD16^+^ apoptotic monocytes. Infliximab reduced the apoptotic CD16^−^ cells. In conclusion, diabetes mellitus type 1 is associated with greater apoptosis of three monocyte subsets which may contribute to the development of microvascular complications. TNF-*α* modifiers appear to ameliorate monocyte apoptosis. They may be useful for controlling excessive monocyte apoptosis in diabetic patients.

## 1. Introduction

Diabetes mellitus type 1 (DM1) is associated with defects in TNF-*α* signaling which result in an altered balance between TNF's prosurvival and proapoptotic effects. One of the manifestations of this is prolonged survival of immune B cells and both CD4^+^ and CD8^+^ T lymphocyte subsets [1, 2]. On the other hand, in patients with DM1 enhanced apoptosis is prevalent, occurring in pancreatic cells, endothelial retinal cells, and various renal tissues [3–5]. The apoptosis defect has also been found in nonobese diabetic (NOD) mice, a spontaneous model of human DM1. Lymphocytes from these animals are more susceptible to TNF-*α*-induced apoptosis than those from control animals [5]. Moreover, apoptosis develops in the liver of streptozotocin-induced diabetic rats and is mediated by the TNF-R1 signaling pathway [6]. Neutrophils from diabetic patients display an enhanced apoptotic activity whereas those from diabetic mice show a reduced apoptotic rate [7, 8].

Molecular studies in NOD mice have revealed several genetic defects that alter the immune function within the MHC genomic region. A point mutation has been localized in the shared promoter-enhancer region of genes encoding LMP2 and TAP-1 proteins, which negatively regulates the transcription of these genes. This mutation impairs proteolytic cleavage and activation of the NF-*κ*B transcription factor, which is physiologically bound to the inhibiting I*κ*-B*α* subunit. NOD mice, therefore, do not show degradation of the I*κ*-B*α* subunit, which permanently suppresses the NF*κ*-B-dependent transcription of cell survival-promoting proteins. Thus, stimulation of lymphocytes by TNF-*α* preferentially triggers the proapoptotic pathway [5, 9, 10].

In humans, the genetic defects that are important for altered apoptosis are distinct from those in NOD mice. An important role has been ascribed to a mutation identified in a gene-coding small ubiquitin-like modifier 4 (SUMO4) [11]. The SUMO4-encoded protein is involved in the ubiquitination of the I*κ*-B*α* subunit. A single amino acid substitution defect was found to prevent the NF*κ*-B-dependent activation. Such negative regulation of the NF*κ*-B transcription factor hampers transcription of the prosurvival and antiapoptotic proteins [11, 12]. Analysis of several clinical studies on different human populations has enabled an association to be found between multiple polymorphism in and near the gene SUMO4 and susceptibility to diabetes mellitus type 1 [13].

Many more molecular defects are expected in DM1, but these have not yet been detailed. Different malfunctioning proteins have already been found in the TNF-R2 signaling pathway, resulting in inefficacies in the TNF-R2 pathway which is important for cell survival and cell proliferation. Thus, in the case of nonactive TNF-R2 receptors, the only way of signaling by TNF-*α* may be through the proapoptotic TNF-R1-dependent path [10]. All these data imply that in diabetic patients, monocytes, cells that are important for innate and specific immune responses, may be vulnerable to apoptosis.

It has been recognized that enhanced apoptosis underlies retinopathy and nephropathy, the late diabetic microvascular complications [4, 5]. TNF-*α*, the chief inflammatory mediator of microangiopathy, triggers a sequence of biological events that culminate in apoptosis of vascular endothelial cells [3]. The exact mechanism of apoptosis of endothelial cells has been understood in retinopathy. In the stages preceding overt retinopathy, the retinal capillary dysfunction is initiated by permanent infiltration by leukocytes. These cells adhere tightly to the endothelial cells as a result of the stimulating effect of TNF-*α* on the expression of adhesive molecules on both endothelial cells and leukocytes. Adhering inflammatory cells produce an array of angiogenic, inflammatory, and fibrogenic factors that promote endothelial cell-junction breakdown, blood-retinal barrier loss, and injury and apoptotic death of retinal endothelium and pericytes [14–16]. Long before overt complications occur in the animal model of diabetes, monocytes form the main constituent of infiltration within the lumen of the retinal microvessels [3]. Similar infiltrations have been found in renal microvessels [16, 17]. A large accumulation of monocytes and granulocytes is responsible for capillary leukostasis, which mechanically blocks blood flow and increases injury [18].

Monocytes appear to be a heterogeneous population. A subset of monocytes, the so called “nonclassical” CD14^+^CD16^+^ monocytes comprise about 10% of the whole CD14^+^ monocyte population. They are enriched in genes associated with the differentiation processes for an antiproliferative and proapoptotic state. The CD16^+^ subsets are expanded in different kinds of inflammatory disease, such as rheumatoid arthritis, Crohn's disease, HIV, hepatitis, severe asthma, coronary artery disease, end-stage renal disease, sarcoidosis, tuberculosis, and stroke [19–22].* In vitro* experiments on whole blood cell cultures have revealed that the CD16^+^ monocytes may be generated by TNF-*α* treatment to approximately 30% of the total monocytes. In the blood of septic patients, the number of these cells correlated with the blood levels of TNF-*α* [23]. The dendritic cells originating from them were better equipped with adhesion molecules, showed properties of migratory cells, and stimulated more strongly the proliferative activity of  TCD4^+^ cells as compared to those originating from classical monocytes [24]. CD16^+^ monocytes produce chemokines that favour their migration to the vascular wall [25]. Thus, they infiltrate capillaries, small veins, and arteries and strongly attach to the endothelial cells [16]. In addition, these cells are chief producers of TNF-*α*, releasing this cytokine preferentially on the layer of endothelial cells and not spilling it over to the blood stream [26].

The second monocyte subset is the “classical” CD14^++^CD16^−^ cells; it constitutes the main monocyte pool, comprising about 85% of the whole monocyte population. They are mobile cells and may be rapidly recruited from the blood to the infected tissues, where they undergo activation and transform into the CD14^+^CD16^+^ forms. When stimulated, these cells produce a set of inflammatory mediators including IL-8, CCL-2, CCL-3, IL-6, TNF-*α*, NO, and ROS [27]. The characteristic clustering of genes found in monocyte subsets indicates that the classical monocytes are highly versatile and capable of responding to a variety of external stimuli and mediating tissue repair and immune functions. They are enriched in genes coding for a proliferative and antiapoptotic state.

Although the distinction between monocyte subsets has been demonstrated in different diseases and in healthy volunteers, there are no data to support the notion that such differentiation also exists among healthy juveniles and DM1 patients. Moreover, bearing in mind the general apoptotic defect in DM1, it would be interesting to characterize the spontaneous apoptotic potential of these monocyte subsets, if it exists. To date no data has appeared concerning the spontaneous apoptotic capacity of monocyte subsets in DM1.

The aim of our study therefore was to provide quantitative data on CD14^++^CD16^−^ and CD14^+^CD16^+^ monocyte subsets in juvenile-onset complication-free diabetes mellitus type 1 and to compare them with the corresponding values in the healthy age- and sex-matched control group. Next, we planned to enumerate the spontaneously apoptotic monocytes within both subsets in the DM1 patients and to compare the results with the values for the healthy subjects. Moreover, since the data imply that the proapoptotic potential of a DM1 patient's cells may be dependent on an overactive TNF-R1 pathway, it would be interesting to determine the expression of TNF-R1 receptors on monocytes and then to manipulate this with TNF-R1 blocking or enhancing agents so that its effect on monocyte apoptosis may be observed. Finally, we wanted to assess the effect of infliximab, which is a chimeric monoclonal antibody against TNF-*α* with a murine variable region and a human immunoglobulin constant region, on the apoptosis of monocytes of DM1 patients and healthy controls.

## 2. Materials and Methods

### 2.1. Participants

The group examined consisted of 60 randomly selected children and adolescents aged 14.5 ± 3.2 years (28 boys and 32 girls) with long-standing DM1 (a disease duration of 5.8 ± 3.6 years, Hba1c = 8.38 ± 2.21%, and an albumin excretion rate of 15.35 ± 7.9) from the Diabetology Outpatient Clinic at the Medical University of Gdańsk, Poland. The diagnosis of DM1 was made in accordance with the American Diabetes Association criteria [28]. Patients with microvascular complications and those with coexisting autoimmune, chronic, and acute inflammatory diseases were excluded from the study. In all the patients, examined C-peptide levels were below 0.5 ng/mL. All patients were treated with humanized insulin. The control group consisted of 30 healthy age- (13.5 ± 2.8 years) and sex-matched (14 males and 16 females) volunteers. Informed consent was obtained from the parents of all the children enrolled in the study, while juveniles over 16 years old consented in person. The study was approved by the Ethics Committee of the Medical University of Gdańsk and was carried out in accordance with the Code of Ethics of the World Medical Association (Declaration of Helsinki 1975 and its amendments 1983, 1989, 1996 JAMA 1997; 277:925-926) for experiments involving humans.

### 2.2. Blood Measurements

HbA_1_c was measured by an immunoturbidometric method using the Unimate 3 set (Hoffmann-La Roche AG, D) with a normal range of 3.0–6.0%. Fasting glucose was measured by an enzymatic test (Roche Diagnostics GmbH, D). Urinary albumin excretion was expressed as the average of three 24-hour collections obtained during the six months prior to enrolment in the study. Microalbuminuria was defined as albumin excretion within the range of 30–299 mg/24 hours in at least two out of three urine samples. Urinary albumin excretion was measured by the immunoturbidometric assay using Tina-quant (Boehringer Mannheim GmbH, D).

### 2.3. Identification of Monocyte Subsets: CD14^++^CD16^−^ and CD14^+^CD16^+^ Monocytes and TNF-R1-Positive Apoptotic Cells

Peripheral blood mononuclear cells were obtained by density gradient centrifugation on the Ficoll/Isopaque (Pharmacia, S). Isolated peripheral blood mononuclear cells were placed on 24-well plates in RPMI 1640 containing 5% FCS (Sigma Aldrich, USA) at a density of 1 × 10^5^/well. Fc receptors were blocked with 1 *μ*g of human IgG/10^5^ cells for 15 minutes at room temperature.

Samples from each patient were divided into three series. The first was left without additives and the second was kept with 50 *μ*L of infliximab (Remicade, Janssen Biologics B.V.; NL). The third set was incubated with one of the following substances: anti-TNF-R1 mAb (*clone H398*) 10 *μ*g/mL (Serotec L.T.D., USA), anti-TNF-R2 mAb* (clone M1)* 10 *μ*g/mL (Serotec L.T.D., USA), 16 *μ*M, sodium salicylate 10 mM, and 4-bromophenacyl bromide (BPB) (Sigma Aldrich, USA). Plates were incubated for 24 h at 37°C with 5% CO_2_. After incubation plates were kept for 5 minutes on ice.

The cell suspension was transferred to cytometric tubes. The tubes were rinsed three times with cold PBS and then centrifuged and the supernatants removed. The sedimented cells were suspended in 200 *μ*L of 5% RPMI and the following mAbs were added: 4 *μ*L anti-CD14 PerCP (*clone MΦP9; IgG2*_*b*_) (BD Biosciences, USA), 3 *μ*L anti-CD16 APC-Cy7 or FITC (*clone 3G8; IgG*_*1*_) (BD Biosciences, USA), and 5 *μ*L anti-TNF-R1 PE (*clone16803; IgG*_*1*_) (R&D Systems, USA). Cells of the healthy volunteer group were divided into only two parts, those without a modifier and those with infliximab.

### 2.4. Enumeration of Apoptotic Monocytes

Parallel with specific staining fluorochrome-conjugated isotypic controls were run. Plates were incubated for 30 minutes in the dark on ice and were then centrifuged and the supernatant was discarded. After being washed with PBS, the cellular pellet was suspended in 70 *μ*L of Annexin V Binding Buffer (BD Biosciences, USA), supplemented with 3 *μ*L of Annexin-FITC (BD Biosciences, USA). Plates were incubated for 15 minutes in the dark at room temperature. After incubation the tubes were filled with 400 *μ*L of Annexin V Binding Buffer and read on a cytometer. The measurements were performed using a LSRII flow cytometer (BD Biosciences, USA) and equipped with a solid state Coherent Sapphire blue laser (20 mW, 488 nm), a solid state UV Laser-light wave X-cyte laser (20 mW, 355 nm), and a helium-neon gas laser (17 mW, 633 nm). The expression of cell surface markers was assessed after gating on live cells determined by scatter characteristics.

### 2.5. Acquisition and Analysis of Flow Cytometry Data

Data were analyzed using the FACS Diva 6.0 Software (Becton Dickinson, USA). The region containing monocytes was generated on the basis of their forward versus right-angle light scatters. Typically, 10,000 events were acquired in this region. CD14 monocytes were assessed using an SSC/CD14 dot plot. The monocyte subsets were analyzed from dot plots representing CD14 versus CD16 staining. The threshold level for the fluorescence of positive cells was set for each sample from a difference between curves obtained from the specific mAb and isotype control mAb staining. TNF-R1-positive cells were analyzed in the CD14PerCP and CD16 FITC gates.

### 2.6. Statistical Analyses

The results were analyzed using Statistica Version 10 (StatSoft, PL). Continuous variables were tested for normality using the Kolmogorov-Smirnov test. Normally distributed variables were analyzed with the ANOVA test. For comparison of the skew-distributed variables the nonparametric Kruskal-Wallis ANOVA test was applied. The significance level was set at *P* < 0.05 and two-sided tests were performed as the standard.

## 3. Results

### 3.1. Monocyte Subsets in the Diabetes Mellitus Type 1 Patients and in the Group of Healthy Subjects

#### 3.1.1. Identification of the Monocyte Subsets

**Figure 1:**
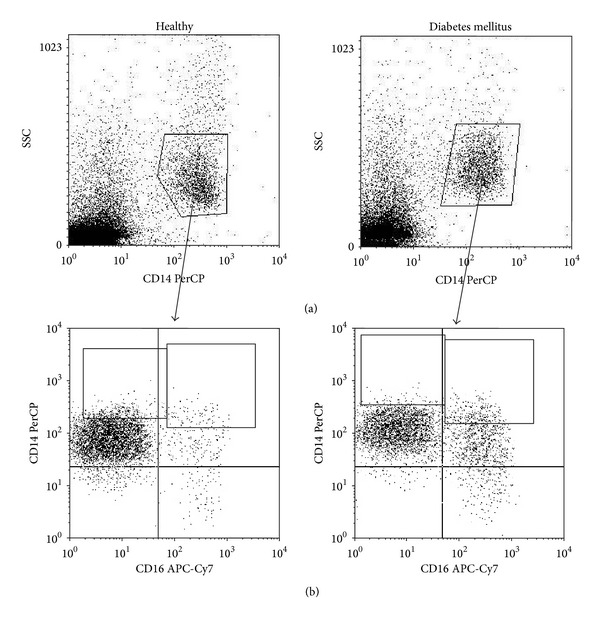
The representative dot plot of monocytes in the healthy and DM1 patients. Monocytes were identified by combination of the SSC parameter and the expression of CD14 molecule (a). Among the CD14^+^ monocytes two subsets were identified on the basis of CD16 receptor expression, the CD14^++^CD16^−^ and CD14^+^CD16^+^ (b).

By means of flow cytometry we identified two monocyte subsets in healthy volunteers and DM1 patients: the CD14^++^CD16^−^ and CD14^+^CD16^+^ cells (Figure 1).

#### 3.1.2. A Comparison of Monocyte Subsets in Patients and in the Healthy Group

The monocytes counts in the DM1 patients and the healthy group are compared in Table 1. The absolute number of CD14^++^CD16^−^ monocytes was significantly lower in DM1 patients as compared with the healthy volunteers. The values of CD14^+^CD16^+^ monocytes showed an inverse trend being higher in the patients than in healthy controls.

#### 3.1.3. The Apoptotic Potential of Monocytes

Since defects of apoptosis in different tissues have been recognized as pathogenic in DM1, the next analysis was a comparison of the apoptotic potential of the DM1 patients' monocytes and those of the healthy subjects. For this purpose the binding of Anexinn V to phosphatidylserine on cell surface was used. Peripheral blood mononuclear cells were incubated for 24 hours in a culture medium. The cells were stained for visualization of the CD14 and CD16 molecules and then with fluorochrome-conjugated Annexin V. The number of apoptotic, Annexin V-positive cells was calculated and is presented in Table 2.

The absolute values and percentages of Annexin V binding cells in DM1 patients were found to be about twice that found in the healthy volunteers (Table 2). This applied to both of the subsets examined. Generally, about half of the cells of the patients' monocytes bound to Annexin V. There were no differences between subsets in this respect.

**Figure 2:**
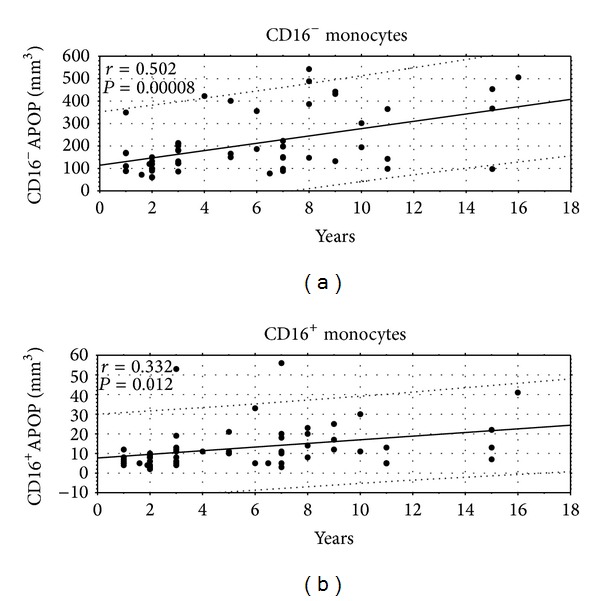
Apoptotic monocyte count in the course of DM1. Apoptotic monocytes were enumerated from the PBMC samples. The relationship between diabetes duration (in years) and absolute values of blood CD16^−^ and CD16^+^ Annexin V binding monocytes was analyzed in sixty diabetic patients. Analysis of Annexin V binding monocytes was carried out in gates determining the CD14 versus CD16 positive monocytes. Coefficient “*r*” and “*P*” values are presented. APOP = apoptotic.

In addition, we observed a significant gradual increase in the pool of CD16^−^ and CD16^+^ apoptotic cells with disease duration (Figure 2).

#### 3.1.4. The Effect of TNF-R1 Receptor Modifiers on Monocyte Apoptosis

The TNF-R1 receptor is recognized as a main mediator of the TNF-*α*-induced apoptotic signal. In DM1 patients, however, defective apoptosis may be associated with an altered signal transduction through the TNF-R2 receptor. The next step was therefore for the factors blocking or augmenting TNF-R1 receptor expression to be added to the incubation medium, and apoptosis was assessed on CD16^−^ and CD16^+^ monocytes.

#### 3.1.5. TNF-R1 Receptor Suppressors Reduce the Apoptotic Capacity of CD16^+^ Monocytes

**Figure 3:**
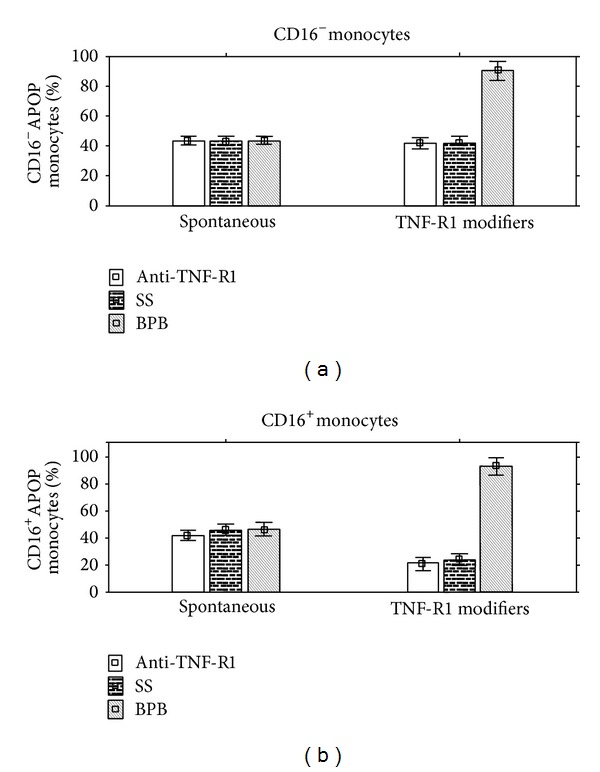
Effect of TNF-R1 modifiers on CD16^−^ and CD16^+^ patients' monocytes apoptosis. Blood was taken from 10 DM1 patients. Peripheral blood mononuclear cells were isolated and incubated for 24 h in culture medium either without additives or with one of the following substances: anti-TNF-R1 mAb (10 *μ*g/mL), 4-bromophenacyl bromide (BPB) (16 *μ*M), and sodium salicylate (SS) (10 mM). After incubation the cells were stained for visualization of CD14, CD16, and Annexin V binding molecules on monocytes. The percentage of CD16^−^ and CD16^+^ apoptotic cells was summarized from 3 experiments and presented as arithmetic mean ± S.D. Statistical significance was calculated with Kruskal-Wallis Anova test. Statistical significance for CD16^−^ monocytes: BPB (*P* = 0.00002); statistical significance for CD16^+^ monocytes: anti-TNF-R1 mAb (*P* = 0.00002), SS (*P* = 0.0002), and PBP (*P* = 0.0002). Anti-TNF-R1: mAb against TNF-R1 receptor; APOP: apoptotic BPB: 4-bromophenacyl bromide; SS: sodium salicylate.

It appeared that anti-TNF-R1 antagonistic mAb significantly decreased the percentage of apoptotic cells only within the CD16^+^ monocyte subset. Sodium salicylate (SS), an irreversible inhibitor of cyclooxygenase (Cox) activity, significantly reduced the percentage of CD16^+^ apoptotic monocytes. Anti-TNF-R2 antagonistic mAb had no significant effect on the parameters examined (data are not presented here) (Figure 3).

#### 3.1.6. TNF-R1 Receptor Potentiating Agent Increases the Apoptotic Capacity of CD16^+^ and CD16^−^ Monocytes

Bromophenacyl bromide (BPB), an agent potentiating apoptosis, increased the percentage of the apoptotic cells in both CD16^−^ and CD16^+^ monocyte subsets by more than 90%. Our results indicate therefore that the TNF-R1 receptor transduces the apoptotic signal in the monocytes of DM1 patients. It is easy to manipulate the apoptotic ability of diabetic monocytes through TNF-R1 modifiers (Figure 3).

### 3.2. The Effect of Infliximab on TNF-R1^+^ Apoptotic CD16^−^ and CD16^+^ Monocytes

**Figure 4:**
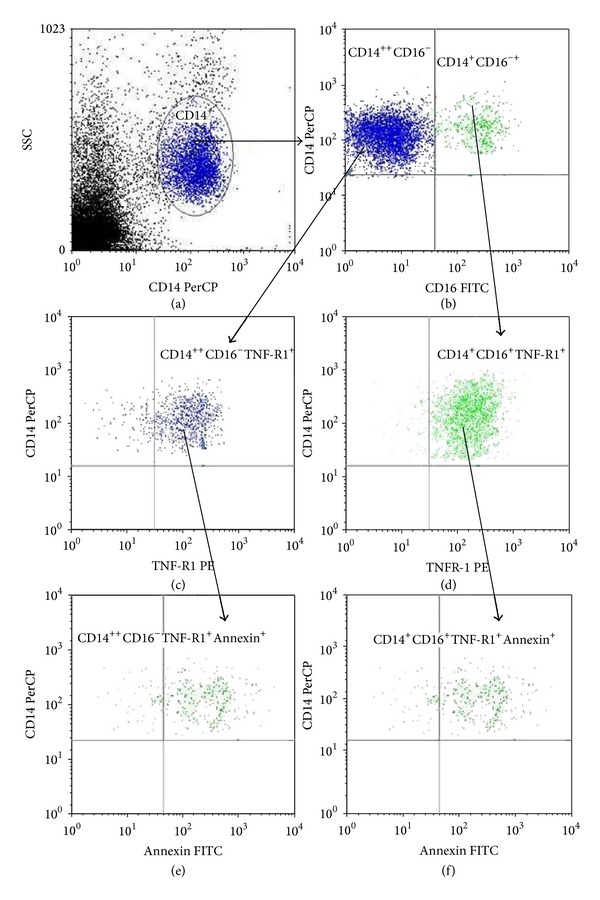
Gating strategy used to analyze apoptosis of monocyte subsets. The monocyte subset was identified according to the expression of the CD14 molecule and SSC parameter (a). Based on the expression of the CD14 and CD16 molecules, two monocyte subsets were distinguished: CD14^++^CD16^−^ and CD14^+^CD16^+^ cells (b). Monocytes in CD14^++^CD16^−^ (c) and CD14^+^CD16^+^ (d) gates were gated on TNF-R1^+^ cells. Plots (e) and (f) show apoptotic Annexin V-positive CD14^++^CD16^−^TNF-R1^+^ and CD14^+^CD16^+^TNF-R1^+^ cells, respectively.

The next stage was to pose the question of whether infliximab, an approved anti-TNF*α* drug, may also control the apoptotic potential of monocytes. The effect of infliximab was analyzed on the TNF-R1^+^ apoptotic monocytes in the DM1 patients and the healthy controls.  Figure 4 shows gating strategy used for analysis of apoptotic TNF-R1^+^ Annexin V-positive monocytes.

**Figure 5:**
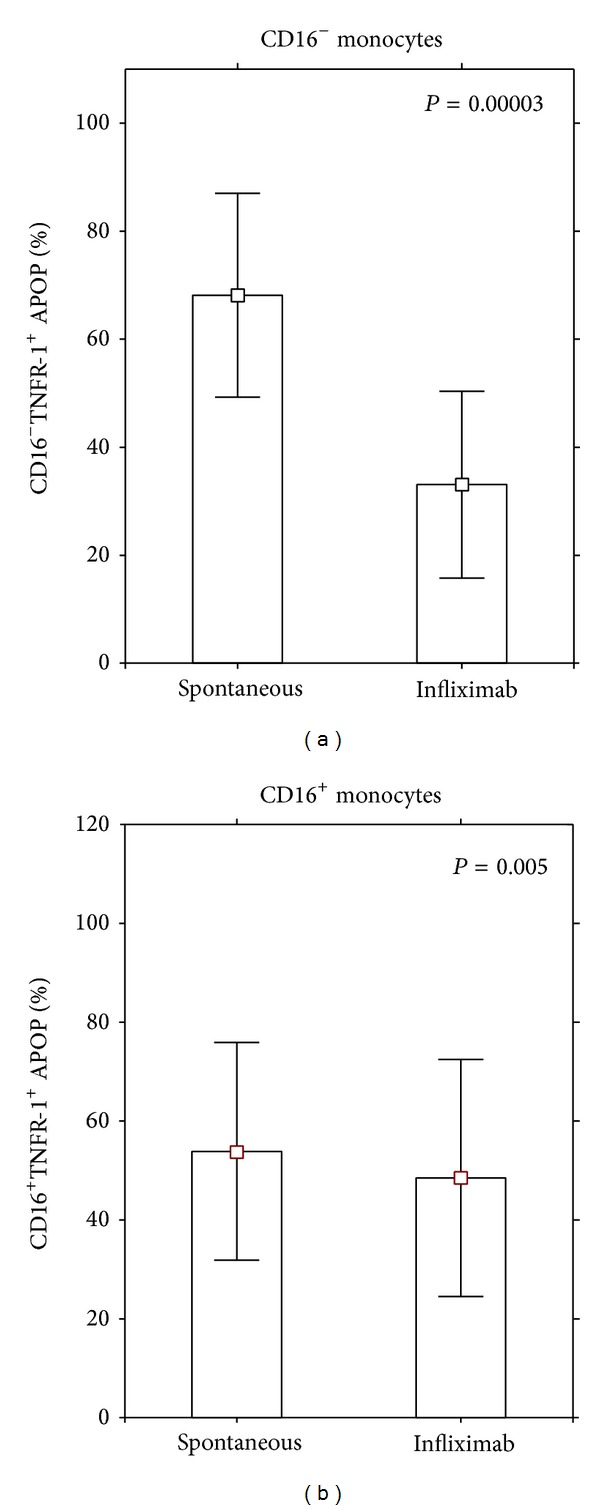
Effect of infliximab on apoptotic CD16^−^ and CD16^+^ TNFR1^+^ monocytes in diabetes mellitus type 1 patients. Peripheral blood mononuclear cells from 60 DM1 patients were incubated for 24 hours in a culture medium and then stained for visualization of the CD14, CD16, and TNF-R1 molecules. Next cellular pellet was incubated for further 15 minutes with fluorochrome-conjugated Annexin V to calculate the number of Annexin V-positive cells. The percentage of CD16^−^ and CD16^+^ TNF-R1^+^ apoptotic cells was summarized and presented as an arithmetic mean ± S.D. Statistical significance was calculated by means of the Kruskal-Wallis Anova test.

Infliximab binds and neutralizes the soluble and transmembrane forms of TNF-*α* and hinders its access to the TNF-R1 and TNF-R2 receptors. Its effect on DM1 patients' monocytes has not been known. In order to learn whether infliximab may influence the apoptotic CD16^−^ and CD16^+^ monocytes, peripheral blood mononuclear cells were incubated in a culture medium for 24 hours either without additives or with 50 *μ*L of infliximab. After incubation the mAb against CD14, CD16, and TNF-R1 were applied together with Annexin V for visualization of the TNF-R1^+^ apoptotic monocytes. The effect of infliximab on the DM1 patients' monocytes is presented in Figure 5.

#### 3.2.1. The Effect of Infliximab on the DM1 Patients' Monocytes

Infliximab reduced the percentage of CD16^−^TNF-R1^+^ apoptotic monocytes while having no effect on the corresponding values of the CD16^+^TNF-R1 apoptotic cells (Figure 5).

#### 3.2.2. The Effect of Infliximab on Monocytes in Subjects in the Healthy Control Group

**Figure 6:**
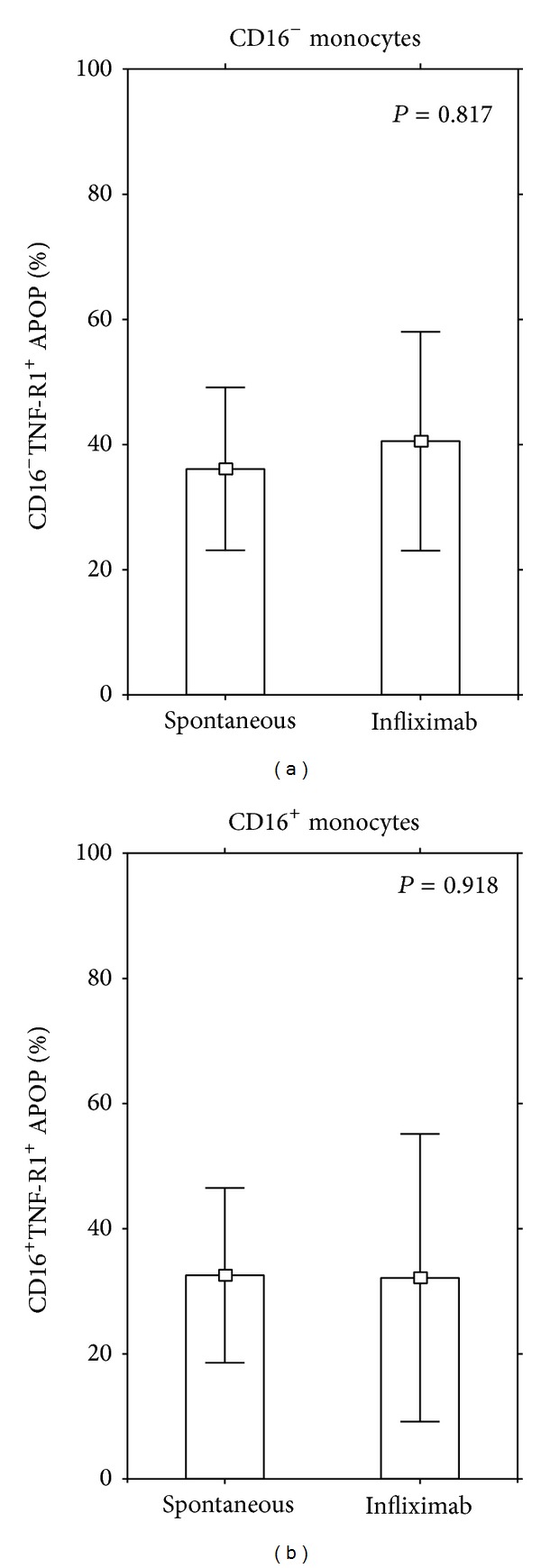
Effect of infliximab on apoptotic CD16^−^ and CD16^+^ TNF-R1^+^ monocytes in the healthy subjects. Peripheral blood mononuclear cells from 30 age- and sex-related healthy were incubated for 24 hours in a culture medium and then stained for visualization of the CD14, CD16, and TNF-R1 molecules. Next cellular pellet was incubated for further 15 minutes with fluorochrome-conjugated Annexin V to calculate the number of Annexin V-positive cells. The percentage of CD16^−^ and CD16^+^ TNF-R1^+^ apoptotic cells was summarized and presented as an arithmetic mean ± S.D. Statistical significance was calculated by means of the Kruskal-Wallis Anova test.

In contrast to its effect on monocytes in the DM1 patients, infliximab did not reduce the percentage of the CD16^−^TNF-R1^+^ and CD16^+^TNF-R1^+^ apoptotic monocytes in the healthy control group (Figure 6).

## 4. Discussion

We found that two monocyte subsets in DM1 patients exhibit an increased spontaneous apoptotic activity* in vitro* in comparison with those in the healthy group. Both subsets showed this defect to the same degree.

Our results complement those already published on enhanced apoptosis affecting various cells and tissues in diabetic patients and diabetes type 1 model animals [3–8]. It seems therefore that enhanced apoptosis is a general phenomenon found in diabetes type 1.

While it is obvious that apoptosis of different cells and tissues poses a serious risk of injury to the organs targeted and of functional failure in these organs, not much is known about the role of apoptotic monocytes in DM1. It can, however, be deduced that apoptotic monocytes cause serious deterioration of the target organs of late diabetic complications. These cells may be causally related to the accelerated development of retinopathy and nephropathy. Apoptotic monocytes may directly stimulate endothelial cell activation and enhance adhesion of blood cells to endothelium. This has been documented in human vascular endothelial cells* in vitro*. This study revealed that the tumor necrosis factor-related apoptosis-inducing ligand (TRAIL)/Apo2 protein, which is responsible for transduction of the TNF-dependent apoptotic signal, simultaneously generates enhanced expression of adhesion molecule ICAM-1 on endothelium in conjunction with enhanced expression of its ligand, the CD18 molecule, on blood cells [29]. The (TRAIL)/Apo2 protein is able to accelerate apoptosis of blood cells, making them adhere firmly to the endothelial wall of small vessels [30]. In this cascade, monocytes that are undergoing apoptosis may exacerbate the process of leukostasis in small renal and eye vessels, directly hindering blood flow and inducing endothelial ischemia. The phagocytic function of the monocytes, which are physiologically responsible for the removal of other apoptotic cells, is also impaired because they die of apoptosis themselves [31]. At the end, TRAIL induces migration of endothelial cell progenitors and, as a consequence, the formation of new vessel tubes, a prerequisite for retinopathy [32]. Greater apoptosis of monocytes thus favors damage to the small blood vessels and promotes angiogenesis in the target organs of microangiopathic diabetic complications.

In order to determine which of the TNF-*α* receptors initiate the apoptotic process, the technique of Annexin V binding was used for enumeration of the apoptotic cells. This technique detects early apoptotic events and enables the TNF-R receptors to be identified on cells in initial phase of apoptosis. Our results therefore indicate that the proapoptotic potential of a DM1 patient's monocytes is dependent on the TNF-R1 pathway. In this paper, we presented that manipulation with drugs that inhibit TNF-R1 expression [33–35] may diminish the pool of CD16^+^ monocytes undergoing apoptosis, while CD16^−^ monocytes appear nonreactive towards TNF-R1 inhibitors. On the other hand, augmentation of apoptosis by PBP [36] reached similar values in both monocyte subsets. This indicates that TNF-*α* modifiers may be useful for controlling excessive monocyte apoptosis in diabetic patients. A reduction in CD16^+^ apoptotic monocytes may be achieved by means of such widely used TNF-R1 blockers as sodium salicylate.

We have recently documented an expansion of the CD16^+^ monocytes in juvenile-onset complication-free DM1 patients during a five-year observation period, during which the percentage and absolute number of the CD14^+^CD16^+^ monocytes doubled [22]. The present study reveals that the expanded monocyte population is not fully functional, since about half entered the early phase of apoptosis.

Our previous study indicated that the size of the nonclassical monocyte subset correlated with the blood level of TNF-*α*, suggesting that this cytokine plays an important role in the expansion of the CD16^+^ monocyte pool. From experiments conducted* in vitro* with monocytes, TNF-*α*-mediated apoptosis of these cells appeared to be regulated by paracrine signaling. TNF-*α* binds to the TNF-R1 receptors, triggering the apoptotic signal. When apoptosis starts the TNF-R1 receptors are still on the monocyte surface, but later the TNF-*α*-TNF-R1 complexes become internalized and the number of TNF-R1-positive monocytes diminishes [37].

Since an enlarged pool of apoptotic monocytes in DM1 patients increases the danger of leukostasis in the small vessels of the target organs of microangiopathy, it would be desirable to reduce the number of these cells in these patients. Our results suggest that TNF-R1 inhibitors such as sodium salicylate and its derivatives may be useful for eliminating the CD16^+^ apoptotic monocytes, while infliximab may be appropriate for the reduction of apoptotic CD16^−^ cells. The two approaches complement each other.

Our experiment with sodium salicylate as TNF-R1-suppressing factor indicates that this widely used anti-inflammatory agent may confer benefits on diabetic patients. Hitherto this substance has not been recommended in DM1 therapy. However, previously published results for NOD mice indicate that sodium salicylate derivatives are effective in protection from retinopathy, which is in keeping with our data [38].

Infliximab has, to our knowledge, not been used in experimental therapy for DM1 patients. However, a recent clinical study indicates the beneficial effect of Etanercept, the other general TNF-inhibitor, on the clinical manifestation of DM1 [39]. Moreover, in clinical studies infliximab has been used successfully with patients with DM1 when this disease coexisted with other autoimmune diseases [40, 41]. In these studies, infliximab appeared to be beneficial for clinical remission of polymyalgia rheumatica as a complication of DM1 [40] and for regression of diabetic sight-threatening macular edema [41]. In animal studies, infliximab inhibited urinary albumin excretion in experimental diabetic rats [42] and ameliorated diabetic neuropathy in mice [43].

In our study, infliximab appeared to moderate apoptosis of monocytes in juvenile DM1 patients. It has been known to neutralize soluble and membrane TNF-*α* and by this means moderates the degree of inflammation. Infliximab causes the TNF-R2 receptors to be shed from monocytes and stimulates interleukin-10 secretion in monocyte cultures* in vitro* [44]. This indicates that infliximab is directing the immune response towards the Th2 profile, a condition required for the effective removal of different apoptotic cells [45]. Moreover, infliximab is able to inhibit inflammation-induced angiogenesis [46] and selectively deplete immature blood vessels [47]. Its putative role in the prevention of microvascular diabetic complications should therefore be further clarified.

It is important to realize that modification of TNF-*α* activity in type 1 diabetes mellitus should be undertaken in complication-free phase of the disease. The study done by group of researches analyzing monocytes in patients with advanced diabetes type 1 and type 2 showed that in patients with late diabetic complications prevails the anti-inflammatory profile with an enhanced IL-10 blood level and CD16^+^ monocytes are at lower frequency as compared with patients without complications. This paper suggests also that the disappearance of CD16^+^ monocytes from blood is associated with their transformation into macrophages [48]. Macrophages probably settle down in tissues and especially on endothelium and become insensitive to action of TNF-*α* modifiers.

In conclusion, diabetes mellitus type 1 is associated with greater apoptosis of monocyte subsets which may contribute to the development of microvascular complications. TNF-R1 modifiers, together with infliximab, appear to ameliorate monocyte apoptosis and should be further examined for their assistance in the treatment of diabetes mellitus as protective agents against late diabetic microvascular complications. They should join other experimental forms of treatment currently under consideration for preventing capillary leukostasis, such as treatment with statins [49], a PPAR*α* agonist [50], or anti-VEGF agents [51].

## Figures and Tables

**Table 1:** Monocyte subsets in diabetes mellitus type 1 patients and healthy group.

	CD14^++^CD16^−^ classical		CD14^+^CD16^+^ nonclassical	
	Absolute values/mm^3^	%	Absolute values/mm^3^	%
Healthy *n* = 30	880 ± 133	96	23.1 ± 0.8	2.54
DM1 *n* = 60	610 ± 150	88	54 ± 17	7.8
Statistical significance *P *	0.0005		0.001	

Values are presented as arithmetic means ± S.D.; DM1—diabetes mellitus type 1.

Statistical differences were calculated between the values for the patients and those for healthy controls within each monocyte subset.

**Table 2:** Apoptotic cells within the monocyte subsets.

	CD14^++^CD16^−^ classical		CD14^+^CD16^+^ nonclassical	
	Healthy	DM1	Healthy	DM1
% of apoptotic cells within subset	22.0 ± 7.1	51.6 ± 21.1	18.5 ± 10.1	45.5 ± 18.2
Statistical significance *P *	0.0000		0.0000	
Absolute number of apoptotic cells/mm^3^	193.8 ± 12.1	314.7 ± 37.4	5.01 ± 0.45	24.6 ± 3.8
Statistical significance *P *	0.0000		0.0000	

Values are presented as arithmetic means ± S.D.; DM1—diabetes mellitus type 1.

Statistical differences were calculated between the values for the patients and those for healthy controls within each monocyte subset.
